# Adverse moisture events predict seasonal abundance of Lyme disease vector ticks (*Ixodes scapularis*)

**DOI:** 10.1186/1756-3305-7-181

**Published:** 2014-04-14

**Authors:** Kathryn A Berger, Howard S Ginsberg, Katherine D Dugas, Lutz H Hamel, Thomas N Mather

**Affiliations:** 1 Center for Vector-Borne Disease, University of Rhode Island, 02881 Kingston, RI, USA; 2 USGS Patuxent Wildlife Research Center, Coastal Field Station, University of Rhode Island, 02881 Kingston, RI, USA; 3 Department of Plant Sciences and Entomology, University of Rhode Island, 02881 Kingston, RI, USA; 4 Department of Computer Science and Statistics, University of Rhode Island, 02881 Kingston, RI, USA; 5 Present address: Faculty of Veterinary Medicine, University of Calgary, GIS Lab TRW 2D28, 3280 Hospital Drive NW, T2N 4Z6 Calgary, AB, Canada

**Keywords:** *Ixodes scapularis*, Relative humidity, Tick activity, Survival

## Abstract

**Background:**

Lyme borreliosis (LB) is the most commonly reported vector-borne disease in north temperate regions worldwide, affecting an estimated 300,000 people annually in the United States alone. The incidence of LB is correlated with human exposure to its vector, the blacklegged tick (*Ixodes scapularis*)*.* To date, attempts to model tick encounter risk based on environmental parameters have been equivocal. Previous studies have not considered (1) the differences between relative humidity (RH) in leaf litter and at weather stations, (2) the RH threshold that affects nymphal blacklegged tick survival, and (3) the time required below the threshold to induce mortality. We clarify the association between environmental moisture and tick survival by presenting a significant relationship between the total number of tick adverse moisture events (TAMEs - calculated as microclimatic periods below a RH threshold) and tick abundance each year.

**Methods:**

We used a 14-year continuous statewide tick surveillance database and corresponding weather data from Rhode Island (RI), USA, to assess the effects of TAMEs on nymphal populations of *I. scapularis*. These TAMEs were defined as extended periods of time (>8 h below 82% RH in leaf litter). We fit a sigmoid curve comparing weather station data to those collected by loggers placed in tick habitats to estimate RH experienced by nymphal ticks, and compiled the number of historical TAMEs during the 14-year record.

**Results:**

The total number of TAMEs in June of each year was negatively related to total seasonal nymphal tick densities, suggesting that sub-threshold humidity episodes >8 h in duration naturally lowered nymphal blacklegged tick abundance. Furthermore, TAMEs were positively related to the ratio of tick abundance early in the season when compared to late season, suggesting that lower than average tick abundance for a given year resulted from tick mortality and not from other factors.

**Conclusions:**

Our results clarify the mechanism by which environmental moisture affects blacklegged tick populations, and offers the possibility to more accurately predict tick abundance and human LB incidence. We describe a method to forecast LB risk in endemic regions and identify the predictive role of microclimatic moisture conditions on tick encounter risk.

## Background

Lyme borreliosis (LB) is the most prevalent vector-borne disease occurring in north temperate regions worldwide, spread by ticks of the genus *Ixodes*[1,2]. To accurately predict disease incidence, and to design effective management programs, it is essential to understand those factors that regulate tick population dynamics. In the case of the blacklegged tick (*Ixodes scapularis*), the primary vector of Lyme disease, babesiosis, and human anaplasmosis in North America, authors have suggested several factors that influence vector survival and, therefore, abundance. Such factors include the population density of white-tailed deer, the primary adult tick reproductive host [3,4], and the population density of disease reservoir rodents [5], which can be influenced by acorn mast production [6] and weather [7,8]. Assessing the effects of weather-related variables can be very complex because of the potentially confounding effects of host-related dynamics, the potential for shifting importance of different population regulating factors from year to year [9], and the difficulty in determining which weather variables actually affect tick survival.

Abundance and LB infection prevalence of questing ticks are important variables in determining disease risk to humans. An entomological index has previously been developed for Rhode Island (RI), USA, providing a measure of infected nymphal *I. scapularis* encountered per unit of time sampled [10]. This index was strongly predictive of LB risk for the state, supporting the hypothesis that case distribution was largely a function of peridomestic risk. However, Mather et al. [10] observed extreme variability of tick abundance in similar habitats, suggesting that additional environmental factors may regulate tick population dynamics. Given their diminutive size, *I. scapularis* nymphs can display sensitivity to conditions of low environmental moisture, with laboratory studies confirming greater nymphal mortality under low humidity conditions [11,12].

Nymphs of *I. scapularis* are more susceptible to desiccation when questing for their next blood meal because of their high surface to volume ratio [11]. Indeed, nymphal ticks can desiccate within 48 h if deprived of moisture, even though they are able to imbibe water moisture from partially saturated air [11,13]. However, field studies on the relationship between environmental moisture, tick populations, and LB incidence, have produced conflicting results. Some investigators have documented associations between climatic variables such as drought and precipitation events with both tick populations [8] and LB incidence [14,15]. In contrast, other studies have found only modest or no relationship between precipitation and tick numbers [5,16]. One plausible explanation for these confounding results is that the weather variables measured did not consistently reflect the environmental conditions experienced by the ticks. Weather station data are generally collected from airport monitoring areas and might not be related in a simple fashion to the microclimatic conditions within the leaf litter habitats where nymphal *I. scapularis* dwell. Furthermore, tick survival might bear a more complex relationship to environmental moisture than previously documented. Indeed, Rodgers et al. [12] reported significant nymphal *I. scapularis* mortality after the ticks were continuously exposed to atmospheric moisture below an 82% relative humidity (RH) threshold for more than 8 hours, even when RH was subsequently increased to favorable levels.

In this study, we present new insight into the mechanism by which environmental moisture regulates blacklegged tick populations and tick encounter risk. This work is the result of a 14-year retrospective analysis of a statewide tick surveillance program and corresponding weather monitoring data in RI between 1997–2010.

## Methods

**Figure 1:**
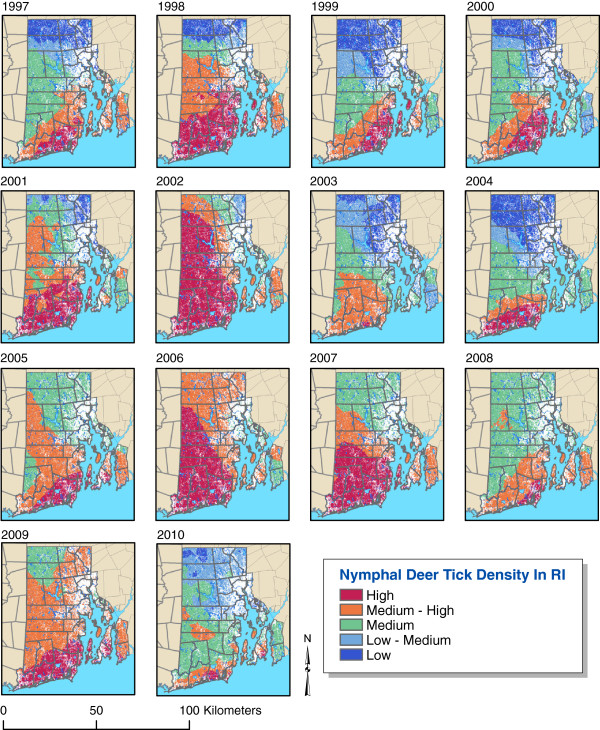
**Inter-annual variability in the seasonal spatial abundance of nymphal *Ixodes scapularis *ticks over several years in the state of Rhode Island.** Map Produced By URI Center For Vector-Borne Disease. Data Sources - RIGIS, URI Center For Vector-Borne Disease.

We examined the relationship between extended periods of sub-threshold atmospheric moisture and tick populations using 14 years of nymphal *I. scapularis* surveillance data collected in RI. The TickEncounter Risk Survey (http://tickencounter.org/multimedia/rhode_island_map), an extensive statewide tick surveillance program focused on nymphal *I. scapularis*, has been in continuous operation since 1993 [17,18]. Although RI is geographically the smallest state in the United States, the nymphal blacklegged tick surveillance database is potentially the most extensive available of the annual abundance of this tick species. This unique database demonstrates a substantial amount of inter-annual variability in nymphal blacklegged tick abundance (Figure 1) despite a well-established presence of this vector within the region.

Samples are collected by drag-sampling from more than 60 forested locations throughout RI. For each sampling location, a 0.5 m^2^ white flannel flag is dragged across the leaf litter at 30 second intervals as described [10]. A total of 90 drags are completed for each location, per sampling round (n = 180 annually). All field sites are sampled twice between late May and August following these standardized sampling protocols. Each year, sites are assigned a random sampling order during Round 1 (late May-late June) and this is repeated in Round 2 (July-August), effectively capturing the entire peak of nymphal tick abundance during their typical summertime seasonal phenology. Forested areas, representing suitable tick habitats were sampled, and simple tick count totals were recorded for each site. Of more than 60 sampling sites currently being visited, the 37 that had been continuously sampled over the entire period were used for this retrospective study.

Hourly RH data were obtained from the National Oceanic and Atmospheric Administration’s (NOAA) National Climatic Data Center (NCDC) Local Climatological Data (LCD) (http://www7.ncdc.noaa.gov/CDO/dataproduct). An interactive graphical user interface (GUI) database processing software system was developed to facilitate downloading and cleaning the NOAA data into a manageable format. To correspond with statewide nymphal *I. scapularis* seasonal abundance totals, all hourly RH data were averaged to generate a single statewide hourly RH database for each year included in the analysis.

**Figure 2:**
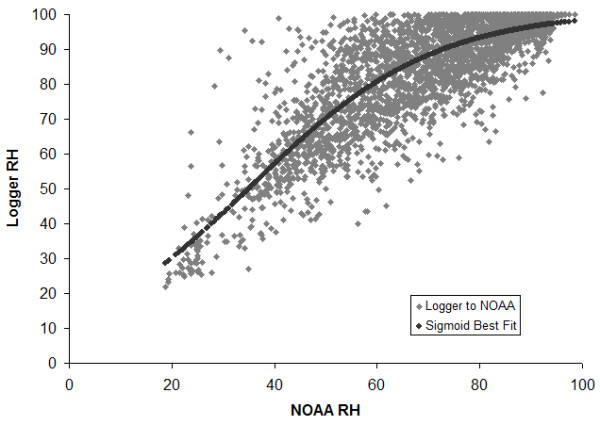
Best fit sigmoid curve predicting RH at loggers in leaf litter from RH recorded at airport weather stations in Rhode Island.

Of the RI weather stations available, three recorded continuous RH observations over the 14-year period (1997–2010) were included in this analysis: (1) Newport State Airport, Newport, RI; (2) Theodore F. Green State Airport, Providence, RI; and (3) Westerly State Airport, Westerly, RI. To determine the RH conditions actually experienced by the ticks, we placed HOBO data loggers (H8 Pro Series, #H08-032-08 Temp/RH Data Loggers, Onset Computer Corp., Bourne, MA) at leaf litter level in 18 RI sites in 2007, and developed a relationship to transform the weather station data into RH values for the leaf litter habitat of the tick [19]. Since the maximum RH of 100% is frequently sustained in leaf litter, the relationship is expected to be asymptotic in nature, so we fit the data to a sigmoidal curve (MatLab, version 7.5.0) of the form: P(x) = K/(1 + e^-x^). The best fit curve (R^2^ = 0.70, P < 0.001) is shown in Figure 2: P(x) = 100/(1 + 7.2145e ^(−0.0559x)^). We used this relationship to quantify the number of tick adverse moisture events (TAMEs) experienced by nymphal ticks in RI each June (the first full month of nymphal activity) during the 14-year study period.

Calculations of TAMEs from hourly NOAA RH data were carried out using a series of if/then statements to determine the total number of sub-82% RH events over a time period > 8 h in duration. All data transformations and TAME calculations were performed in Microsoft Excel (2010). All statistical analyses were performed using SAS 9.2 (SAS Institute, Cary, NC, USA). Sub-optimal RH events occurring in typical tick habitats each year were analyzed as predictors of the total seasonal abundance of nymphal blacklegged ticks in RI between 1997–2010.

## Results

**Figure 3:**
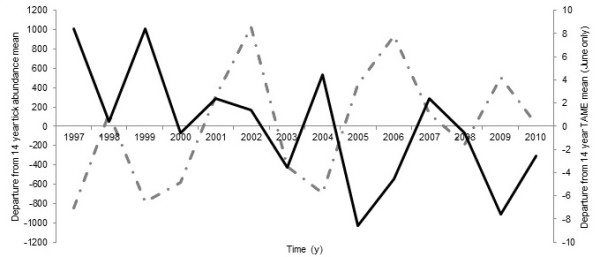
**Time series plot of departures from a 14-year average of nymphal blacklegged tick abundance and June TAMEs recorded for Rhode Island, 1997–2010.** Dashed line represents departure from a 14-year tick abundance mean. Solid line represents departure from a 14 year average of June TAMEs.

**Figure 4:**
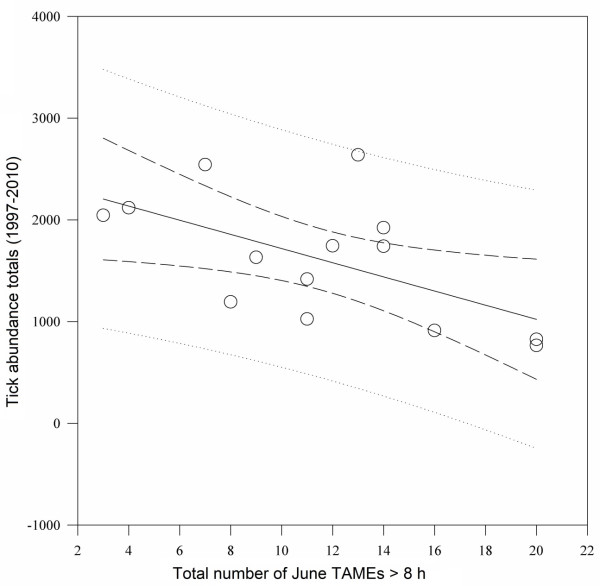
**Linear regression of seasonal nymphal *Ixodes scapularis *** **abundance totals recorded across 37 continuously drag-sampled study areas versus total number of TAMEs (>8 h) recorded in June of the same year (1997–2010).**

Mean total annual tick abundance sampled across all sites (1997–2010) was 1,608. Total number of TAMEs recorded during June of those years averaged 11.6 events (Table 1). Departures from the 14-year averages of both TAMEs and tick densities (Figure 3) indicated that years characterized by a greater number of TAMEs in June generally resulted in below average seasonal totals for nymphal *I. scapularis* abundance. Linear regression of the total number of TAMEs recorded during June successfully predicted total seasonal nymphal tick abundance recorded for the same year (coefficient = −69.57, SE = 27.66, *P* = <0.027) (Figure 4).

**Table 1:** **June TAMEs reduce seasonal abundance values of nymphal *Ixodes scapularis***

**Year**	**Total ticks sampled per year**	**Total June TAMEs (>8 h)**
1997	765	20
1998	1744	12
1999	825	20
2000	1025	11
2001	1922	14
2002	2638	13
2003	1193	8
2004	912	16
2005	2044	3
2006	2541	7
2007	1740	14
2008	1417	11
2009	2118	4
2010	1631	9
*Median*	*1685.5*	*11.5*
*Mean*	*1608.2*	*11.6*
*Standard Deviation*	*589.99*	*4.98*

Relative seasonal abundance values of nymphal *Ixodes scapularis* determined in 37 continuously drag-sampled study sites distributed across Rhode Island and total TAMEs (>8 h) recorded by Rhode Island NOAA stations in June of the same year (1997–2010).

**Figure 5:**
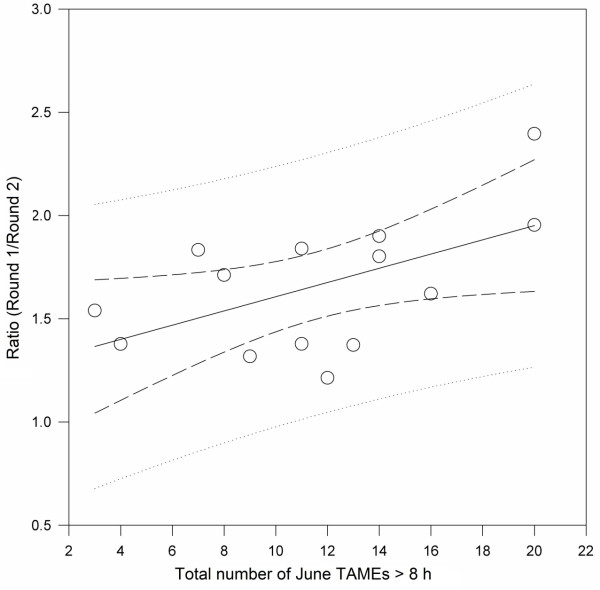
**Linear regression of the ratio of nymphal *Ixodes scapularis *** **collected during round one and round two sampling campaigns across 37 continuously drag-sampled study areas versus total number of TAMEs (>8 h) recorded in June (during round one) of the same year (1997–2010).**

To determine whether the predictive value of low humidity events resulted from tick mortality, we compared early season to late season tick collections on an annual basis. The ratio of ticks collected in Round 1/Round 2 was positively related to the number of TAMEs in June (coefficient = 0.0344, SE = 0.015, *P* = 0.040) (Figure 5).

Multiple regression of a four-parameter model incorporating degree of winter severity (defined by total days below 32°C) recorded between 1997 and 2010, failed to significantly improve model function (*P* = 0.660). The three additional variables added to the simple linear model were: (1) TAMEs (>8 h) for June two years prior, (2) total days below 32°C during the prior winter, and (3) total days below 32°C two winters prior. These additional parameters examined the role of June moisture availability and winter severity on the two-year life cycle of *I. scapularis,* but did not significantly improve the analysis.

## Discussion

TAMEs likely have their greatest impact on ticks questing above or nearer the top of the leaf litter surface. The few estimates available suggest that roughly 10% of the total *I. scapularis* population in leaf litter is questing at any given time [20]. If, however, TAMEs occur early and often enough in the seasonal phenology of the tick, they may deplete >10% of the questing population. If TAMEs induced tick mortality, the number of TAMEs should predict the ratio of the collections in Round 1 to those in Round 2. Our data suggest that an accumulation of TAMEs in June led to significantly lower seasonal nymphal *I. scapularis* abundance totals in some years. Likewise, years with below average nymphal *I. scapularis* abundance in RI (1997, 1999, 2000, 2003, 2004, 2008) tended to have higher than average numbers of TAMEs during June (Figure 3).

We quantified TAMEs using a single threshold of 82% RH at typical RI summer temperatures to simplify our analysis. However, laboratory studies suggest that even lower ambient RH levels, and longer durations of below-threshold RH levels, may result in more severe effects on tick survival [12]. Quantification of the effects on tick survival over a broad range of values for environmental moisture, and for duration of below-threshold conditions, could be used to refine predictions of the effects of extended periods of desiccation on nymphal blacklegged tick populations.

The importance of accumulated time periods of desiccating conditions for tick survival apparently also applies to tick species other than *I. scapularis*. For example, ‘high-saturation deficit events’ were associated with increased tick mortality and reduced questing periods of *I. ricinus* in the suburban forests of Neuchâtel, Switzerland [21]. Simple models providing insight into the duration of questing behavior of *I. ricinus* ticks as a result of immediate environmental conditions have been developed previously [22]; however, few studies have been able to elucidate the relationship between readily available environmental parameters and seasonal population dynamics [23,24]. Individual tick species differ considerably in their tolerance of low humidity levels, so studies would be needed for each tick species to determine threshold RH levels, and population-level responses to specific desiccating conditions. The results presented in this study are specific for *I. scapularis* of RI, but may be applicable for adjacent LB endemic areas with similar climatic conditions. The validation of this model to the rest of New England and Mid-Atlantic states would be necessary to further confirm our findings.

Other factors governing nymphal tick availability during sampling may include host density patterns, which were not accounted for in this retrospective study and may explain some of the observed noise in Figure 3. Additionally, temperature has a profound effect on RH and may help to explain the behavioral activity of questing nymphs [25], but it was not included in this retrospective analysis. However, the results of our study clarify the association between environmental moisture and tick survival by demonstrating a significant relationship between TAMEs and tick abundance each year. Previous studies have not considered (1) the differences between relative humidity (RH) in leaf litter and at weather stations, (2) the RH threshold that affects nymphal blacklegged tick survival, and (3) the time required below the threshold to induce mortality. We suggest that by accounting for these factors, we are able predict total tick abundance for the year by calculating the number of moisture limiting events early in the life cycle of the tick.

We envision the TAMEs identified in this study could be effectively incorporated in a weather-based forecast system of tick encounter exposure for endemic populations. Public warnings could be improved and targeted during periods of greatest risk (e.g. fewer TAMEs events during early and mid-summer). Knowledge of how to protect oneself when in tick habitats would be better targeted during periods of greatest tick encounter exposure. Additionally, preventative measures such as perimeter spraying could be better timed to more effectively reduce questing nymph populations by incorporating our added knowledge on TAMEs and their influence on tick survival. Lastly, we propose that this validation of a laboratory-identified RH threshold on tick survival could be incorporated into the framework of models predicting the effects of climate change on tick encounter risk.

## Conclusion

Our results provide new insight into the means by which environmental moisture influences tick survival. Previous studies [5,8,15,16] have had variable success at predicting tick population densities or tick-borne disease incidence. Potentially confounding effects may have been due to the use of weather data from remote weather stations where RH values are not linearly related to RH in tick habitats (Figure 2), or the application of broad measures of environmental moisture, such as long-term values for RH or precipitation. Ticks do not respond to these factors directly because of a physiological threshold, below which survival is affected. Selected climatic variables are often too coarse to capture the specific component affecting tick survival: extended periods of below-threshold desiccating conditions.

Previous studies that have demonstrated weak relationships between climate variables and tick abundance have utilized coarse candidate climate variables such as total growing season precipitation in the current year [5] or Palmer Hydrologic Drought Index, PHDI [14]. Our results suggest that these variables do not capture the moisture limiting dynamics, biologically relevant to tick survival, that occur on a daily basis, and only provide weak associations to total tick abundance. The TAMEs reported in our study provide a variable that is both biologically relevant and temporally sensitive to modeling human exposure to LB.

Current climate change models predict increased precipitation extremes [26], which would result in periods of high environmental moisture as well as periods of drought. Our results suggest that these changes will have profound effects on *I. scapularis* populations. More accurate predictability of within-season tick population trends will allow better targeted public education and tick control efforts, so as to more efficiently mitigate the growing public health impact of tick-borne diseases.

## Abbreviations

RH: relative humidity; TAME: Tick adverse moisture event; LB: Lyme borreliosis; RI: Rhode island; NOAA: National oceanic and atmospheric administration; NCDC: National climatic data center; LCD: Local climatological data; GUI: Graphical user interface; PHDI: Palmer hydrologic drought index, PHDI.

## Competing interests

The authors declare that they have no competing interests.

## Authors’ contributions

KB cleaned data, performed quality control of data, analyses and wrote paper. KD performed RH-NOAA sigmoid function analysis. LH supervised NOAA GUI development. HG was involved in scientific discussions and data analysis, and edited paper. TM conceived and supervised project, edited paper. All authors discussed the results and commented on and approved the final version of the manuscript.
